# Chemical Composition and Antibacterial Activity of Essential Oils from the Algerian Endemic *Origanum glandulosum* Desf. against Multidrug-Resistant Uropathogenic *E. coli* Isolates

**DOI:** 10.3390/antibiotics9010029

**Published:** 2020-01-15

**Authors:** Larbi Zakaria Nabti, Farida Sahli, Hocine Laouar, Ahmed Olowo-okere, Joice Guileine Nkuimi Wandjou, Filippo Maggi

**Affiliations:** 1 Laboratoire de Valorisation des Ressources Biologiques Naturelles (LVRBN), Université de Sétif 1, Sétif 19000, Algeria; sahlimicrobio@yahoo.fr (F.S.); h-laouar@gmail.com (H.L.); 2 Département des Sciences Naturelles, École Normale Supérieure Assia DJEBAR, Constantine 25000, Algeria; 3 Department of Pharmaceutics and Pharmaceutical Microbiology, Usmanu Danfodiyo University Sokoto, Sokoto 840244, Nigeria; olowoahmed@gmail.com; 4 School of Pharmacy, University of Camerino, 62032 Camerino, Italy; joice.nkuimiwandjou@unicam.it

**Keywords:** multidrug-resistant *E. coli*, *Origanum glandulosum* Desf., essential oil, GC-MS, antibacterial activity

## Abstract

Antibiotics are becoming ineffective against resistant bacteria. The use of essential oils (EOs) may constitute an alternative solution to fight against multidrug-resistant bacteria. This study aims to determine the chemical composition of EOs from five populations of the endemic Algerian *Origanum glandulosum* Desf. and to investigate their potential antibacterial activity against multidrug-resistant uropathogenic *E. coli* strains. The EOs were obtained by hydrodistillation and their composition was investigated by gas chromatography/mass spectrometry (GC/MS). The antibacterial activity was evaluated by the disc diffusion method against eight *E. coli* strains (six uropathogenic resistant and two referenced susceptible strains). Minimum inhibitory and bactericidal concentrations (MIC/MBC) were obtained by the broth microdilution method. The main EO components were thymol (15.2–56.4%), carvacrol (2.8–59.6%), *γ*-terpinene (9.9–21.8%) and *p*-cymene (8.5–13.9%). The antibacterial tests showed that all the EOs were active against all the strains, including the multidrug-resistant strains. The EO from the Bordj location, which contained the highest amount of carvacrol (59.6%), showed the highest antibacterial activity (inhibition diameters from 12 to 24.5 mm at a dilution of 1/10). To our knowledge, this is the first description of the activity of *O. glandulosum* EOs against resistant uropathogenic strains. Our study suggests that *O. glandulosum* EO could be used in some clinical situations to treat or prevent infections (e.g., urinary tract infections) with multidrug-resistant strains.

## 1. Introduction

Urinary tract infections (UTIs) are ones of the main hospital- and community-acquired bacterial infections [1]. *Escherichia coli,* the microorganism that is the most frequent cause of UTIs, has become resistant to many available antibiotics, including third generation cephalosporins, carbapenems and colistin [1,2]. Infections caused by multidrug-resistant (MDR) bacteria have become a major healthcare problem worldwide [3,4]. Currently, there is a great need to search for new, natural molecules that have antibacterial effects and that can act against these MDR strains. Essential oils (EOs), also known as volatile oils, are one of the main natural bioactive substances extracted from plants, and have been used as alternative medicines, especially as antimicrobial agents [5]. Among them, the EOs obtained from the genus *Origanum* L. have attracted the attention of microbiologists due to their widespread use as natural food preserving agents [6].

*O. glandulosum* Desf. (synonymous with *O*. *vulgare* subsp. *glandulosum* (Desf.) Ietswaart) is a member of the *Lamiaceae* family and an endemic plant in two African-Mediterranean countries, namely, Algeria and Tunisia [7,8]. Local populations use this plant for its medicinal properties to treat different diseases such as cough, fever and bronchitis [8]. Several studies have reported the chemical composition of Algerian [7,8,9,10,11,12] and Tunisian [13,14] *O. glandulosum* EOs and showed that thymol, carvacrol, *γ*-terpinene and *p*-cymene were the main components. However, the antibacterial properties of *O. glandulosum* EOs have only been examined in a few studies, mainly, against reference strains of the American Type Culture Collection (ATCC) [7,14,15]. To the best of our knowledge, there have been no reports on the antibacterial properties of *O. glandulosum* EOs against MDR-uropathogenic *E. coli* strains. Thus, the aims of this study were: (i) to determine the chemical composition of EOs extracted from five different populations of *O. glandulosum* growing in Algeria, and (ii) to investigate the potential antibacterial activity of these EOs against MDR-uropathogenic *E. coli* strains.

## 2. Results

### 2.1. Yield and Chemical Composition of the Essential Oils

The obtained EO yields were 3.9, 5.6, 4.1, 1.8 and 2.8% (w/w) for accessions from Sétif, Mila, Bordj, M’sila and El Oued, respectively. As shown in Table 1, the gas chromatography/mass spectrometry (GC/MS) analysis identified 43 (EO from M’sila), 37 (EO from Sétif), 37 (EO from Bordj), 34 (EO from Mila) and 21 (EO from El Oued) components, respectively, which accounted for 99.8–100% of the total composition of the EOs. For all the EOs, the four main detected components were thymol (15.1–56.3%), carvacrol (2.8–59.6%), *γ*-terpinene (9.8–21.8%) and *p*-cymene (8.5–13.9%). Thymol was predominant in EOs from Sétif (56.3%), Mila (51.0%) and M’sila (40.1%), while carvacrol was the major component in EOs from Bordj (59.6%) and El Oued (45.1%).

### 2.2. Antibacterial Activity

The disc diffusion, minimum inhibitory concentration (MIC) and minimum bactericidal concentration (MBC) results (Table 2 and Table 3) showed that all the EOs exhibited good antibacterial activity against both reference (*E. coli* ATCC 25922 and *E. coli* J53) and MDR *E. coli* strains. The MDR *E. coli* 229, *E. coli* 667 and *E. coli* 115 strains were less susceptible than the other strains, while the standard *E. coli* ATCC 25922 strain was the most susceptible. When the EOs were diluted at 1/2, the inhibition zones of all the EOs were ≥23 mm, while the positive control gentamycin (disc charge = 15 µg) was less effective, and there were no inhibition zones against some strains or halos produced with diameters < 23 mm. Carvacrol and thymol were the most active components, and showed good antibacterial activity (the MIC/MBC ranged from 0.25 to 0.5 mg/mL) (Table 2 and Table 3). *p*-Cymene was less active (inhibition zone diameters between 10 and 12.5 mm at a dilution of 1/2 and MIC/MBC > 2 mg/mL), while *γ*-terpinene was totally inactive against all the strains (Table 2). Combinations of *p*-cymene + carvacrol and *p*-cymene + thymol showed very similar results (inhibition zone diameters between 28 and 32 mm).

## 3. Discussion

In this study, we investigated the chemical composition and the antibacterial activity of *O. glandulosum* EOs obtained from five different Algerian locations. The five EOs shared a similar qualitative composition, which was mainly characterized by four major components (thymol, carvacrol, *γ*-terpinene and *p*-cymene). Our findings were qualitatively in agreement with those reported in the literature [7,8,9,10]. However, the composition of our five EOs differed quantitatively. Thymol was the major component in three EOs (Sétif, Mila, M’sila), while carvacrol was predominant in the two remaining ones (Bordj, El Oued). Several studies have reported that carvacrol [7,8,9,10,14] was the major component in *O. glandulosum* EOs, while other studies on the chemical composition of *O. glandulosum* EOs collected from other localities, showed that thymol was the predominant component [7,10,15]. In another study, Khalfi et al. [12] reported comparable amounts of both compounds (thymol 38.8% and carvacrol 32.9%). Conversely, Mechergui et al. [13] found that *p*-cymene (36%, 40% and 46%) followed by thymol (32%, 39% and 18%) were the major components detected in *O. glandulosum* EOs obtained from three localities (Nefza, Bargou and Krib, respectively) in the north of Tunisia.

Although all the previous studies (including our study) involved the same plant species (*O. glandulosum*), we observed differences in the chemical composition, especially in the quantity of the main EO components. This might be due to variations in many factors that affect the investigated accessions, such as climate and temperature, type of soil, relief, period of plant collection and the part of the plant (e.g., leaves, stems and flowers) [16]. In our study, all the five accessions were collected during the same period. Moreover, the same parts of plants were used to obtain the EOs. However, with regard to the climate, the M’sila and El-Oued areas are characterized by high temperatures and low precipitation rates whereas Sétif and Mila are located in rocky regions and at higher altitude.

The evaluation of the antibacterial activity of the five EOs against six uropathogenic MDR and two standard *E. coli* strains revealed that all the five EOs were active against all the tested strains, showing similar inhibition zone diameters as well as MIC and MBC values. The EO from Bordj contained the highest amount of carvacrol (59.6%) and showed the highest antibacterial activity (diameters from 12 to 24.5 mm at dilution of 1/10). To our knowledge, this is the first description of the antibacterial properties of *O. glandulosum* EOs against MDR uropathogenic *E. coli* strains. Béjaoui et al. [14] reported the good activity of Tunisian *O. glandulosum* EOs tested at different phenological stages against a standard *E. coli* strain (*E. coli* ATCC 8739). Similar results (good growth inhibition) were also obtained when testing different Algerian *O. glandulosum* EOs against *E. coli* ATCC 25922 (standard strain) [7] and three other clinical strains (E_1_, E_2_ and E_3_) [9].

EOs containing high amounts of phenolic monoterpenes, such as carvacrol and thymol are widely reported to have remarkable antibacterial properties [17]. These compounds are capable of aligning between the fatty acid chains to form a sort of channel through the membrane, and are able to interact with transmembrane proteins, thus affecting the microbial cell permeability. They also exert detrimental effects on the outer membrane of Gram-negative bacteria [18]. Another important microbial effect reported for these phenolic monoterpenes is their interference with the ATP generation system [18].

Since our five EOs were rich in thymol and/or carvacrol, we suggest that the obtained antibacterial activities were related to the high concentration of these two constituents. It is assumed that these compounds act by damaging the structure and function of the cytoplasmic membrane [19]. However, it has also been reported that the antibacterial activity of EOs could be affected by other minor components, together with a possible interaction between different compounds [14]. For example, *p*-cymene may synergize the efficacy of phenolic monoterpenes such as thymol and carvacrol [20]. *p*-Cymene is highly hydrophobic and causes swelling of the cytoplasmic membrane, thus facilitating the transport of effective active compounds across the lipid bilayer. Our results counteract previous reports on the antibacterial activity of *p*-cymene [20], proving that it is an effective agent against uropathogenic MDR strains, and could possibly be used in synergistic blends together with phenolic monoterpenes. These are relatively cheap, available on the market and easy to prepare, thus making it possible to produce formulations against multidrug-resistant uropathogenic *E. coli* strains where cultivation of *O. glandulosum* and production of its EOs is not feasible.

## 4. Materials and Methods

### 4.1. Plant Material

The *O. glandulosum* Desf. samples were collected on June 2018 from 5 different locations situated in the east and southeast of Algeria: Chirhom (Sétif city), Sidi Aissa (M’sila city), Ras El oued (Bordj-Boarriridj city), Bouslah (Mila city) and Ben Guecha (El Oued city). The plants were identified by Prof. Laouar Hocine, University of Sétif-1, Algeria and confirmed by Prof. Filippo Maggi.

### 4.2. Essential Oil Extraction

The air-dried aerial parts (stems, leaves, and flowers) of *O. glandulosum* Desf. were submitted to hydrodistillation for 3 h using a Clevenger-type apparatus. The obtained EOs were collected in amber glass vials and stored at 4 °C until used for analysis.

### 4.3. GC/MS Analysis

The chemical analysis of the different EOs was carried out using an Agilent 6890 N gas chromatograph, coupled with a 5973 N mass spectrometer, operating in the EI mode at 70 eV, using a HP-5MS capillary column (5% phenylmethylpolysiloxane, 30 m, 0.25 mm i.d., 0.1 μm film thickness) (J & W Scientific, Folsom, CA, USA). The temperature schedule that was applied for this analysis was as follows: 60 °C for 5 min, followed by a 4 °C/min ramp to 220 °C, then 11 °C/min up to 280 °C, hold for 15 min and finally 11 °C/min up to 300 °C, hold for 5 min. The carrier gas used was helium, at a flow rate of 1.0 mL/min. The temperature of the injector and transfer line was 280 °C with an injection volume of 2 µL and a split ratio of 1:50. The scan time was 75 min and the acquisition range mass spectra *m*/*z* 29–400. The identification relied on the combination of linear retention indices (RIs) and mass spectra (MS) with those stored in Adams, FFNSC2 and NIST17 libraries [21,22,23]. Relative peak area percentages were extracted for each peak from the total area in the chromatograms without using correction factors.

### 4.4. Bacterial Strains

The *E. coli* strains were isolated at the microbiology laboratory of Sétif University Hospital, Algeria from patients with urinary tract infections. Identification of the strains was performed by matrix-assisted laser desorption ionization time of flight Mass Spectrometry (MALDI-TOF MS) (Bruker Daltonics, Bremen, Germany). Four *E. coli* strains (*E. coli* 734, 854, 292, 229) were resistant to 3rd and 4th generation cephalosporins and produce extended spectrum β-lactamase (ESBL) enzymes [1]. One strain (*E. coli* 667) was resistant to both cephalosporins and carbapenems (ertapenem) by producing ESBL and OXA-48 enzymes [1], and one other strain (*E. coli* 115) was resistant to colistin, which is considered as the last resort treatment of carbapenem resistant *Enterobacteriaceae* [2]. Two reference strains (*E. coli* ATCC 25922 and *E. coli* J-53) were also used for the antibacterial tests.

### 4.5. Screening for Antibacterial Activity

The disc-diffusion method was used to investigate the potential antibacterial activity of the EOs against six uropathogenic MDR and two reference susceptible *E. coli* strains. A culture of 0.5 McFarland from each strain was seeded on Muller Hinton (MH) agar plates using a sterile swab. Sterile blank paper discs of 6 mm diameter (Becton Dickinson, Le Pont de Claix, France) were gently pressed onto the MH agar plates. EOs (15 µL) and pure components (carvacrol, thymol, *p*-cymene and *γ*-terpinene, Sigma-Aldrich, Milan, Italy) (15 µL/14 mg) were pipetted onto the blank discs and each EO was tested pure and at two other dilutions (1/2 and 1/10) in DMSO. Blank discs impregnated with DMSO and discs of gentamycin (15 µg; SIRScan Discs, i2a, Montpellier, France) were used as negative and positive controls, respectively. Plates were incubated for 18–24 h at 37  °C and the diameter of the inhibition zones was measured. According to Ponce et al. [24], sensitivity was classified as: not sensitive (diameter <8 mm), sensitive (diameter of 9–14 mm), very sensitive (diameter of 15–19 mm) and extremely sensitive (diameter >20 mm).

### 4.6. Determination of MIC and MBC

The MIC and MBC were determined in 96-well plates (12 columns and 8 rows). For each EO, 10 different concentrations were tested (70, 35, 17.5, 8.75, 4, 2, 1, 0.5, 0.25, 0.125 mg/mL) (columns 12 to 3, respectively). Negative (MH broth and DMSO) and positive (MH broth and bacterial inoculum, without essential oils) controls were prepared for each plate in columns 1 and 2, respectively. One *E. coli* strain was tested in each row (8 strains tested from row A to H). The plates were incubated overnight at 37 °C. The MIC values corresponded to the first well of each row where no visible bacterial growth was detected. The MBC was determined from the first three wells of each row that showed no bacterial growth after plate incubation. For that, 10 µL from the corresponding wells were seeded on MacConkey agar (Becton Dickinson, Le Pont de Claix, France) plates. After overnight incubation at 37 °C, any bacterial growth was checked. The MBC values represent the concentrations from plates where no bacterial colonies were found.

### 4.7. Statistical Analysis

All experiments were performed in duplicate and data were expressed as mean values ± standard deviation (SD).

## 5. Conclusions

In summary, thymol, carvacrol, *γ*-terpinene and *p*-cymene were the main components found in the different *O. glandulosum* EOs, with a predomination of thymol in EOs from Sétif, Mila and M’sila and carvacrol in EOs from Bordj and El Oued. All EOs were active against all *E. coli* strains including the MDR ones. The EO from the Bordj location contained the highest amount of carvacrol (59.6%), and showed the highest antibacterial activity (diameters from 12 to 24.5 mm at dilution of 1/10). *O. glandulosum* EOs have potential use as natural antibacterial agents to treat or prevent infections (e.g., urinary tract infections) with MDR strains. The effectiveness of the *O. glandulosum* EOs has been shown to strictly depend on the content of carvacrol, thymol and *p*-cymene. Thus, where it is not possible to cultivate the plant in order to obtain active EOs, the possibility of manufacturing artificial blends containing carvacrol, thymol and *p*-cymene could be considered. Further studies are still necessary to assess the safety of these EOs for clinical use.

## Figures and Tables

**Table 1:** Chemical composition of the EOs from five populations of the Algerian endemic *O. glandulosum* Desf.

Component ^a^	RI Calc. ^b^	RI Lit. ^c^	EO %				
			Sétif	Mila	Bordj	M’sila	El Oued
(2*E*)-Hexenal	846	846	^d^ tr	-	0.1	0.1	-
3-Heptanone	883	890	tr	-	-	tr	-
α-Thujene	918	924	1.2	1.0	0.7	0.5	0.4
α-Pinene	923	932	0.5	0.4	0.4	0.4	0.4
Camphene	936	946	tr	tr	tr	tr	-
Sabinene	962	969	tr	tr	tr	-	-
β-Pinene	965	974	0.1	0.1	0.1	0.1	tr
1-Octen-3-ol	972	974	0.1	0.2	0.2	0.4	0.2
3-Octanone	982	979	tr	0.1	0.1	0.1	tr
Myrcene	985	988	1.4	1.2	0.8	1.0	0.7
3-Octanol	994	988	-	-	-	tr	-
α-Phellandrene	1000	1002	0.2	0.2	0.1	0.1	0.1
δ-3-Carene	1005	1008	tr	tr	tr	tr	-
α-Terpinene	1011	1014	2.6	2.2	1.2	1.7	1.4
*p*-Cymene	1019	1025	9.7	10.3	8.5	13.9	11.6
Sylvestrene	1022	1032	0.4	0.3	0.2	0.3	0.2
(*Z*)-β-Ocimene	1034	1032	tr	tr	tr	tr	-
(*E*)-β-Ocimene	1044	1044	tr	tr	tr	tr	-
*γ*-Terpinene	1053	1054	21.8	19.4	9.8	14.2	13.4
*cis*-Sabinene hydrate	1061	1065	tr	tr	tr	tr	-
(2Z)-Hexenal diethyl acetal	1079	1081	-	tr	-	tr	-
Terpinolene	1082	1086	tr	tr	tr	tr	-
*p*-Cymenene	1084	1089	-	-	tr	tr	-
*trans*-Sabinene hydrate	1093	1098	tr	tr	tr	tr	-
Linalool	1097	1095	0.6	0.7	0.6	0.7	0.3
Borneol	1158	1165	tr	tr	0.1	0.1	tr
Terpinen-4-ol	1170	1174	0.3	0.2	0.3	0.4	0.1
*p*-Cymen-8-ol	1182	1179	tr	tr	tr	0.1	-
α-Terpineol	1185	1186	0.3	0.3	0.4	0.4	0.1
*cis*-Dihydro carvone	1192	1191	-	-	tr	tr	-
*trans*-Dihydro carvone	1200	1200	-	-	tr	-	-
Thymol, methyl ether	1231	1232	0.1	0.1	tr	0.2	0.1
Carvacrol, methyl ether	1240	1241	tr	tr	0.2	0.1	0.1
Carvenone	1258	1255	tr	-	-	tr	-
Thymol	1295	1289	56.3	51.0	15.1	40.1	25.3
Carvacrol	1301	1298	2.8	10.9	59.6	23.4	45.1
Thymol acetate	1350	1349	tr	-	-	tr	-
Carvacrol acetate	1367	1370	-	-	-	tr	-
(*E*)-Caryophyllene	1403	1417	1.2	0.8	0.6	0.7	0.3
*trans-*α-Bergamotene	1424	1432	-	-	-	tr	-
α-Humulene	1436	1452	tr	tr	tr	tr	-
β-Bisabolene	1495	1505	0.1	0.1	0.2	0.4	tr
δ-Cadinene	1507	1522	-	-	-	tr	-
β-Sesquiphellandrene	1509	1521	0.2	0.2	0.3	0.1	-
Caryophyllene oxide	1562	1582	tr	0.1	0.1	0.2	-
Grouped compounds (%)							
Monoteropene hydrocarbons			37.9	35.1	21.9	32.2	28.2
Oxygenated monoterpenes			60.4	63.2	76.2	65.2	71.1
Sesquiterpene hydrocarbons			1.4	1.1	1.2	1.3	0.3
Oxygenated sesquiterpenes			tr	0.1	0.1	0.2	-
Others			0.3	0.4	0.6	0.9	0.4
Number of identified compounds			37	34	37	43	21
Total identified (%)			99.9	99.9	99.9	99.8	100.0

^a^ Order of components according to their elution from a HP-5MS column. ^b^ RI calc.: calculated retention index. ^c^ RI lit.: retention index reported from the literature. ^d^ tr: trace.

**Table 2:** Antibacterial activity of the EOs from five populations of the endemic Algerian *O. glandulosum* expressed as diameter inhibition zones.

City of Collection	EO Dilution	Inhibition Diameters (mm) ± SD							
		Bacterial Strains Tested (Resistance Phenotypes)							
		*E. coli* 734 (ESBL)	*E. coli* 854 (ESBL)	*E. coli* 292 (ESBL)	*E. coli* 229 (ESBL)	*E. coli* 667 (ESBL + CRB)	*E. coli* 115 (COL R)	*E. coli* ATCC 25922	*E. coli* J53
Sétif	Pure	39.6 ± 0.77	34.8 ± 0.56	36.0 ± 1.34	31.2 ± 0.56	31.0 ± 1.41	32.1 ± 0.98	38.4 ± 0.84	38.2 ± 0.28
	1/2	36.7 ± 0.56	33.5 ± 0.98	35.4 ± 0.63	31.0 ± 1.34	27.9 ± 0.56	30.7 ± 1.55	37.1 ± 0.98	36.4 ± 1.97
	1/10	14.6 ± 0.28	15.4 ± 0.28	15.6 ± 0.14	13.9 ± 00	12.0 ± 00	13.9 ± 0.56	17.9 ± 1.41	22.0 ± 00
Mila	Pure	38.5 ± 2.12	36.3 ± 0.7	34.9 ± 0.56	30.6 ± 2.47	32.6 ± 1.27	30.9 ± 0.42	39.6 ± 1.97	38.4 ± 1.69
	1/2	37.4 ± 0.84	35.4 ± 0.28	33.1 ± 0.63	29.5 ± 0.56	30.9 ± 0.56	28.9 ± 0.98	37.4 ± 00	37.6 ± 0.28
	1/10	-	15.4 ± 0.84	-	-	11.9 ± 0.42	12.4 ± 0.28	18.6 ± 00	22.8 ± 0.84
Bordj	Pure	37.7 ± 0.56	35.5 ± 1.83	35.1 ± 0.98	32.6 ± 0.28	27.1 ± 00	26.3 ± 0.84	38.4 ± 0.84	37.6 ± 0.77
	1/2	36.9 ± 00	34.0 ± 00	33.7 ± 0.56	30.1 ± 2.12	24.4 ± 0.28	25.6 ± 1.41	38.2 ± 00	35.6 ± 0.28
	1/10	17.2 ± 1.13	16.4 ± 0.56	17.3 ± 0.84	16.2 ± 00	12.1 ± 0.98	12.0 ± 0.28	21.8 ± 0.84	24.5 ± 1.83
M’sila	Pure	36.3 ± 1.55	35.5 ± 00	35.1 ± 0.98	32.1 ± 0.63	28.2 ± 0.28	24.6 ± 1.27	32.0 ± 0.28	36.2 ± 0.56
	1/2	35.9 ± 1.27	32.3 ± 0.84	33.8 ± 00	29.9 ± 0.28	24.9 ± 0.42	23.6 ± 0.42	31.0 ± 0.28	32.4 ± 0.56
	1/10	15.0 ± 0.14	14.7 ± 0.28	15.5 ± 0.84	12.9 ± 00	10.8 ± 1.13	12.8 ± 00	12.8 ± 0.84	17.3 ± 0.84
El Oued	Pure	36.8 ± 0.28	32.6 ± 1.41	36.2 ± 00	30.4 ± 0.56	26.7 ± 0.56	26.3 ± 0.7	35.7 ± 1.55	34.6 ± 1.27
	1/2	34.8 ± 0.84	32.3 ± 0.98	35.4 ± 0.56	28.5 ± 1.69	24.8 ± 0.28	23.0 ± 1.55	32.4 ± 00	32.7 ± 0.56
	1/10	14.8 ± 00	13.1 ± 0.98	16.7 ± 0.56	-	-	-	-	-
**Pure compounds**									
carvacrol	Pure	36.0 ± 00	34.0 ± 0.7	34.5 ± 0.7	30.0 ± 00	31.5 ± 0.7	32.5 ± 0.7	36.5 ± 0.7	36.5 ± 0.7
	1/2	30.5 ± 0.7	30.0 ± 00	31.0 ± 00	28.5 ± 0.7	30.0 ± 00	30.5 ± 0.7	31.0 ± 00	30.5 ± 0.7
	1/10	28.0 ± 00	27.0 ± 00	28.5 ± 0.7	26.0 ± 00	26.0 ± 00	27.0 ± 00	28.0 ± 00	28.0 ± 00
thymol	Pure	34.5 ± 07	33.0 ± 00	34.0 ± 00	29.0 ± 00	30.0 ± 0.0	32.0 ± 00	35.0 ± 00	36.0 ± 00
	1/2	30.0 ± 00	30.0 ± 00	30.0 ± 0.7	28.0 ± 00	29.0 ± 00	30.0 ± 00	30.5 ± 07	31.0 ± 00
	1/10	27.0 ± 00	27..0 ± 00	28.0 ± 0.7	26.5 ± 07	26.0 ± 00	27.0 ± 00	28.0 ± 00	28.0 ± 00
*p*-cymene	Pure	14.0 ± 00	14.0 ± 00	14.5 ± 0.7	12.0 ± 00	13.5 ± 0.7	14.0 ± 00	14.0 ± 00	14.0 ± 00
	1/2	12.5 ± 0.7	12.0 ± 00	11.5 ± 0.7	10.0 ± 00	10.0 ± 00	12.0 ± 00	12.0 ± 00	11.5 ± 0.7
	1/10	10.0 ± 00	9.0 ± 00	10.0 ± 00	-	-	8.0 ± 00	10.0 ± 00	10.0 ± 00
*γ*-terpinene	Pure	-	-	-	-	-	-	-	-
	1/2								
	1/10								
carvacrol + *p*-cymene	50/50 (%) (*v*/*v*)	30.0 ± 00	30.0 ± 00	30.0 ± 00	29.0 ± 00	30.0 ± 00	30.0 ± 00	30.5 ± 0.7	31.0 ± 00
thymol + *p*-cymene	50/50 (%) (*v*/*v*)	30.0 ± 00	30.0 ± 00	30.0 ± 00	28.0 ± 00	29.0 ± 00	30.0 ± 00	32.0 ± 00	31.0 ± 00
Gentamycin (+control)		18.7 ± 0.21	-	-	17.3 ± 0.84	-	18.6 ± 0.63	21.4 ± 0.77	22.7 ± 0.91
DMSO (-control)		-	-	-	-	-	-	-	-

ESBL: extended spectrum β-lactamase, CRB: carbapenemase producer, COL R: colistin resistant, DMSO: dimethyl sulfoxide, SD: standard deviation, v: volume, (-): no activity observed.

**Table 3:** Minimum inhibitory concentration (MIC) and minimum bactericidal concentration (MBC) of *O. glandulosum* EOs and main active pure compounds.

*O. glandulosum* Collection Site	MIC (MBC) mg/mL							
	*E. coli* 734	*E. coli* 854	*E. coli* 292	*E. coli* 229	*E. coli* 667	*E. coli* 115	*E. coli* ATCC 25922	*E. coli* J53
Sétif	1 (1)	2 (2)	2 (2)	2 (2)	2 (2)	2 (2)	1 (1)	1 (1)
Mila	2 (2)	2 (2)	2 (2)	2 (2)	2 (2)	2 (2)	1 (1)	2 (2)
Bordj	1 (2)	2 (2)	1 (1)	2 (2)	2 (2)	2 (2)	1 (1)	1 (1)
M’sila	1 (1)	1 (1)	1 (1)	2 (2)	1 (1)	1 (1)	1 (1)	1 (1)
El Oued	1 (2)	2 (2)	2 (2)	2 (2)	1 (2)	2 (2)	1 (1)	2 (2)
Major bioactive components								
carvacrol	0.25 (0.25)	0.5 (0.5)	0.25 (0.5)	0.5 (0.5)	0.5 (0.5)	0.5 (0.5)	0.25 (0.25)	0.25 (0.25)
thymol	0.25 (0.25)	0.5 (0.5)	0.5 (0.5)	0.5 (0.5)	0.5 (0.5)	0.5 (0.5)	0.25 (0.25)	0.25 (0.25)
*p*-cymene	>2	>2	>2	>2	>2	>2	>2	>2
Gentamycin mg/L	2	>4	>4	2	>4	2	1	1

## References

[1] Nabti L.Z., Sahli F., Radji N., Mezaghcha W., Semara L., Aberkane S., Lounnas M., Solassol J., Didelot M.-N., Jean-Pierre H. (2019). High Prevalence of Multidrug-Resistant Escherichia coli in Urine Samples from Inpatients and Outpatients at a Tertiary Care Hospital in Sétif, Algeria. Microb. Drug Resist..

[2] Nabti L.Z., Sahli F., Hadjadj L., Ngaiganam E.P., Lupande-Mwenebitu D., Rolain J.-M., Diene S.M. (2019). Autochthonous case of mobile colistin resistance gene mcr-1 from a uropathogenic Escherichia coli isolate in Sétif Hospital, Algeria. J. Glob. Antimicrob. Resist..

[3] Rai J., Randhawa G.K., Kaur M. (2013). Recent advances in antibacterial drugs. Int. J. Appl. Basic Med. Res..

[4] Nabti L.Z., Sahli F., Ngaiganam E.P., Radji N., Mezaghcha W., Lupande-Mwenebitu D., Baron S., Rolain J.-M., Diene S.M. (2019). Development of real-time PCR assay allowed describing the first clinical Klebsiella pneumoniae isolate harboring plasmid-mediated colistin resistance mcr-8 gene in Algeria. J. Glob. Antimicrob. Resist..

[5] Man A., Santacroce L., Jacob R., Mare A., Man L. (2019). Antimicrobial Activity of Six Essential Oils Against a Group of Human Pathogens: A Comparative Study. Pathogens.

[6] Stefanakis M.K., Touloupakis E., Anastasopoulos E., Ghanotakis D., Katerinopoulos H.E., Makridis P. (2013). Antibacterial activity of essential oils from plants of the genus *Origanum*. Food Control.

[7] Sari M., Biondi D.M., Kaâbeche M., Mandalari G., D’Arrigo M., Bisignano G., Saija A., Daquino C., Ruberto G. (2006). Chemical composition, antimicrobial and antioxidant activities of the essential oil of several populations of Algerian *Origanum glandulosum* Desf. Flavour Fragr. J..

[8] Belhattab R., Larous L., Figueiredo A.C., Santos P.A.G., Barroso J.G., Pedro L.G. (2005). *Origanum glandulosum* Desf. grown wild in Algeria: Essential oil composition and glycosidic bound volatiles. Flavour Fragr. J..

[9] Bendahou M., Muselli A., Grignon-Dubois M., Benyoucef M., Desjobert J.-M., Bernardini A.-F., Costa J. (2008). Antimicrobial activity and chemical composition of *Origanum glandulosum* Desf. essential oil and extract obtained by microwave extraction: Comparison with hydrodistillation. Food Chem..

[10] Ruberto G., Baratta M.T., Sari M., Kaâbeche M. (2002). Chemical composition and antioxidant activity of essential oils from Algerian *Origanum glandulosum* Desf. Flavour Fragr. J..

[11] Berrehal D., Boudiar T., Hichem L., Khalfallah A., Kabouche A., Al-Freihat A., Ghannadi A., Sajjadi E., Mehrabani M., Safaei-Ghomi J. (2010). Comparative composition of four essential oils of Oregano used in Algerian and Jordanian folk medicine. Nat. Prod. Commun..

[12] Khalfi O., Sahraoui N., Bentahar F., Boutekedjiret C. (2008). Chemical composition and insecticidal properties of *Origanum glandulosum* (Desf.) essential oil from Algeria. J. Sci. Food Agric..

[13] Mechergui K., Coelho J.A., Serra M.C., Lamine S.B., Boukhchina S., Khouja M.L. (2010). Essential oils of Origanum vulgare L. subsp. glandulosum (Desf.) Ietswaart from Tunisia: Chemical composition and antioxidant activity. J. Sci. Food Agric..

[14] Béjaoui A., Chaabane H., Jemli M., Boulila A., Boussaid M. (2013). Essential oil composition and antibacterial activity of Origanum vulgare subsp. glandulosum Desf. at different phenological stages. J. Med. Food.

[15] Benbelaid F., Khadir A., Abdoune M.A., Bendahou M., Muselli A., Costa J. (2014). Antimicrobial activity of some essential oils against oral multidrug-resistant Enterococcus faecalis in both planktonic and biofilm state. Asian Pac. J. Trop. Biomed..

[16] Macedo da Silva R.O., Gonçalves Castro J.W., de Menezes Dantas Junior O., Justino de Araújo A.C., do Nascimento Silva Leandro M.K., Oliveira Costa R.J., Leite Pinto L., Garcia Leandro L.M., da Silva L.E., do Amaral W. (2019). Photoinduced Antibacterial Activity of the Essential Oils from Eugenia brasiliensis Lam and Piper mosenii C. DC. by Blue Led Light. Antibiotics.

[17] Chouhan S., Sharma K., Guleria S. (2017). Antimicrobial Activity of Some Essential Oils-Present Status and Future Perspectives. Medicines.

[18] Hyldgaard M., Mygind T., Meyer R.L. (2012). Essential oils in food preservation: Mode of action, synergies, and interactions with food matrix components. Front. Microbiol..

[19] Sikkema J., de Bont J.A., Poolman B. (1995). Mechanisms of membrane toxicity of hydrocarbons. Microbiol. Rev..

[20] Burt S. (2004). Essential oils: Their antibacterial properties and potential applications in foods—A review. Int. J. Food Microbiol..

[21] Adams R. (2007). Identification of Essential Oil Components by Gas Chromatography/Mass Spectrometry.

[22] FFNSC 2 (2012). Flavors and Fragrances of Natural and Synthetic Compounds.

[23] NIST 17 (2017). Mass Spectral Library (NIST/EPA/NIH).

[24] Ponce A.G., Fritz R., del Valle C., Roura S.I. (2003). Antimicrobial activity of essential oils on the native microflora of organic Swiss chard. LWT Food Sci. Technol..

